# Single Nucleotide Polymorphisms in IL17A and IL6 Are Associated with Decreased Risk for Pulmonary Tuberculosis in Southern Brazilian Population

**DOI:** 10.1371/journal.pone.0147814

**Published:** 2016-02-03

**Authors:** Mariana Milano, Milton Ozório Moraes, Rodrigo Rodenbusch, Caroline Xavier Carvalho, Melaine Delcroix, Gabriel Mousquer, Lucas Laux da Costa, Gisela Unis, Elis Regina Dalla Costa, Maria Lucia Rosa Rossetti

**Affiliations:** 1 Programa de Pós-Graduação em Biologia Celular e Molecular, Universidade Federal do Rio Grande do Sul, Porto Alegre, Rio Grande do Sul, Brazil; 2 Centro de Desenvolvimento Científico e Tecnológico, Fundação Estadual de Produção e Pesquisa em Saúde, Porto Alegre, Rio Grande do Sul, Brazil; 3 Laboratório de Hanseníase, Instituto Oswaldo Cruz Fundação Oswaldo Cruz, Rio de Janeiro, Rio de Janeiro, Brazil; 4 Division of Infectious Disease and Vaccinology, University of California, Berkeley, United States of America; 5 Universidade Federal de Ciências da Saúde de Porto Alegre, Porto Alegre, Rio Grande do Sul, Brazil; 6 Programa de Pós-Graduação em Clínica Médica, Universidade Federal do Rio de Janeiro, Rio de Janeiro, Rio de Janeiro, Brazil; 7 Hospital Sanatório Partenon, Porto Alegre, Rio Grande do Sul, Brazil; 8 Universidade Luterana do Brazil, Canoas, Rio Grande do Sul, Brazil; Public Health Research Institute at RBHS, UNITED STATES

## Abstract

In *Mycobacterium tuberculosis* (MTB) infection, the complex interaction of host immune system and the mycobacteria is associated with levels of cytokines production that play a major role in determining the outcome of the disease. Several single-nucleotide polymorphisms (SNPs) in cytokine genes have been associated with tuberculosis (TB) outcome. The aim of this study was to evaluate the association between previously reported SNPs *IL2–*330 T>G (rs2069762); *IL4*–590 C>T (rs2243250); *IL6*–174 G>C (rs1800795); *IL10*–592 A>C (rs1800872); *IL10*–1082 G>A (rs1800896); *IL17A* -692 C>T (rs8193036); *IL17A* -197 G>A (rs2275913); *TNF* -238 G>A (rs361525); *TNF* -308 G>A (rs1800629) and *IFNG* +874 T>A (rs2430561) and pulmonary TB (PTB) susceptibility. We conducted a case-control study in individuals from Southern Brazil who were recruited between February 2012 and October 2013 in a high incidence TB city. We performed a multiplex genotyping assay in 191 patients with PTB and 175 healthy subjects. Our results suggest a decreased risk for PTB development associated with the *IL17A* -197A allele (OR = 0.29; p = 0.04), AA genotype (OR = 0.12; p = 0.04) and A carrier (AG/AA) (OR = 0.29; p = 0.004) and *IL6* -174C carrier (CC/CG) (OR = 0.46; p = 0.04). We could not properly analyze *IL17A* -692 C>T (rs8193036) and *IFNG* +874T>A due to genotypic inconsistencies and found no evidence of association for the *IL2*, *IL4*, *IL10* and *TNF* polymorphisms and PTB. In conclusion, our results show a protective effect of *IL17* and *IL6* polymorphisms on PTB outcome in Southern Brazilian population.

## Introduction

Tuberculosis (TB) is a chronic granulomatous disease that predominantly affects the lungs and is caused by *Mycobacterium tuberculosis* (MTB) [1]. TB is ranked as the second leading cause of death from a single infectious agent, and remains a public health issue with 1.5 million of deaths in 2013 [2]. Annually 5.7 million of new TB cases are reported worldwide. Disturbingly, according to World Health Organization approximately one-third of the world’s population is infected with MTB [2].

Consistent with a long lasting host–pathogen interaction and co-evolution, only 10% of MTB-infected individuals progress towards active disease [3]. The mechanisms explaining why some individuals develop the disease are unclear [4]. A significant proportion of interindividual variation in susceptibility to TB can be attributed to environmental factors such as malnutrition, but a substantial part has been credited to host genetic factors [5,6]. Strong evidence derived from family-based, twin and segregation genetic studies indicate that the inherited background influences TB outcome [7–9]. The critical importance of CD4+ T cell mediated immunity [10,11] and of the interleukin (IL)-12/interferon-gamma pathway in resistance to MTB has been duly described [12,13]. Unexpectedly, genome-wide association studies (GWAS) have shown a limited success finding either genes or pathways associated with TB. These studies were unable to consistently pinpoint major genes and there are no independent replications in the described chromosome regions: 8q, 11p13 and 18q11 [14–17]. Nevertheless, genetic determinants of TB susceptibility in case-control studies and in some cases followed by meta-analysis confirmed single nucleotide polymorphisms (SNPs) mostly in immunity-related genes. Studies based on such approaches have properly identified polymorphisms in candidate genes such as *IFNG*, *SLC11A1*, *IL10*, *MCP1*, and *P2RX7* [18–23] to be associated with TB susceptibility. For example, *IFNG* +874T>A the presence of the T allele is associated with TB resistance and higher expression of IFN-gamma [18].

In fact, cytokines play a key role in driving the appropriate immune response to mycobacteria via activation of inflammatory and immunomodulatory networks orchestrated by both macrophages and T cells [24]. The interleukin (IL)-17A is a potent pro-inflammatory cytokine capable of inducing chemokine expression, migration of neutrophil and recruitment and trafficking of Th1 cells to parenchymal tissue during TB infection [24, 25]. IL-17A has also been suggested to be critical in the first steps of TB and granuloma formation [24, 25] along with TNF [26]. Likewise, cytokines as IL-2 and IL-6 are known to work together to promote an immune response satisfactory against MTB [27, 28].

Few studies have investigated the influence of polymorphic variants in *IL6* and *IL17A* genes and PTB outcome showing inconsistent results. The aim of our study was to investigate whether *IL6*–174 G>C SNP and *IL17A* -197 G>A polymorphism and additional cytokine genes involved in immune response to MTB (*IL2*, *IL4*, *IL10*, *IFNG* and *TNF*) are associated with genetic susceptibility to pulmonary TB (PTB). In this study, the genetic association of polymorphic variants within cytokine genes was evaluated in a case-control study in a PTB population from Southern Brazil.

## Materials and Methods

### Case–control study

This case-control study recruited 191 PTB patients in a reference hospital for TB in Porto Alegre, the capital of Rio Grande do Sul State in southern Brazil between February 2012 and October 2013.

We enrolled patients > 18 years old, diagnosed with PTB who were on anti-TB and anti-multidrug-resistant (MDR)-TB treatment during any time between disease diagnosis and the completion of treatment. The information about patients’ medical conditions as results of drug susceptibility test, coexisting of forms of extra-pulmonary TB, diabetes, asthma were extracted through medical records. Serum samples were tested for HIV 1/2 and HCV. Patients were confirmed to have PTB by chest X-ray, sputum smear microscopy and culture according recommendations described in Brazilian National TB guideline [29]. Smear sputum samples were tested for MDR-TB at a referral laboratory in Porto Alegre, Brazil [30]. In this study, patients with comorbidities including diabetes, asthma, HCV, HIV or HCV+HIV were included in PTB group and comprised 22% of patients population. Although, some of the comorbidities are known risk factors for TB outcome such as HIV and diabetes, a comparison of patient population stratified according to the presence and absence of comorbidities were not statistically different (data not shown) indicating that comorbidities are unlikely confounding factors. Adjustments in the regression logistic model using comorbidities were not feasible because this condition was present only in patients. We excluded patients who were not in eligible age criteria (≤17 y/o), coexisting of forms of extra pulmonary TB, drug users, prisoners and Native Americans.

The control group was composed by 175 unrelated healthy individuals, over 18 years old, among them 50 household contacts and 125 blood donors recruited at the Hemocentro do Estado do Rio Grande do Sul in Porto Alegre, which is located at the same geographical area that the PTB cases were recruited. All individuals in control group were HIV and HCV or HIV+HCV negatives, without signs or symptoms of TB, previous history of TB or have completed anti-TB prophylaxis. Ethnic background was determined by self-identification as white, non-white, or other. No individual of auto referred Asiatic ancestry participated in the study. All subjects were BCG vaccinated at birth that was confirmed by the presence of the BCG scar.

### Ethics considerations

The study was approved by Research Ethics Committee of Fundação Estadual de Produção e Pesquisa em Saúde, FEPPS-RS #09⁄2011 in Porto Alegre, Rio Grande do Sul, Brazil. An information sheet describing the purpose of the study and the individuals’ rights as participants study were read along, and a copy provided, for all volunteers. The researcher also provided a contact phone number for any additional information about the research. After all clarification, a written informed consent was obtained for each person.

### SNP genotyping

Genomic DNA was isolated from peripheral blood using Nucleo Spin^®^ Blood kit (Macherey-Nagel Inc.) according to the manufacturer´s instructions. Amplification of regions flanking SNPs in cytokines genes *IL2*–330 T>G (rs2069762); *IL4*–590 C>T (rs2243250); *IL6*–174 G>C (rs1800795); *IL10*–592 A>C (rs1800872); *IL10*–1082 G>A (rs1800896); *IL17A* -197 G>A (rs2275913); *IL17A* -692 C>T (rs8193036); *TNF* -238 G>A (rs361525); *TNF* -308 G>A (rs1800629) and *IFNG* +874 T>A (rs2430561) was accomplished with a multiplex PCR on 5 to 10 ng genomic DNA, 0.2 μM of each primer (S1 Table) and Qiagen Multiplex PCR Master Mix (QIAGEN,Invitrogen). Amplification reaction consisted of 95°C for 15 minutes followed by 30 cycles of 35 seconds at 94°C, 90 seconds at 57°C, 90 seconds at 72°C and a final extension for 10 minutes at 72°C. Amplicons were purified using Illustra^™^ EXOProStar^™^ (GE HealthCare^®^) according to the user’s manual recommendation.

SNP analysis were performed on the SNaPshot^®^ Multiplex System ABI Prism (Applied Biosystems^®^, São Paulo, Brazil). SNaPshot primers are listed in S1 Table.

According instructions of user's manual the reactions were performed in a final volume of 10 μL, containing 3.0 μL (0.01 to 0.4 ρmol) of purified multiplex PCR product, 5.0 μL of SNaPshot Multiplex Ready Reaction Mix and 1μl of a pooled of the ten SNaPshot primers described at S1 Table. All the ten primers used in the pooled SNaPshot reactions were previously premixed to have a concentration of 1 μM for each primer. Multiplex single base extension was carried out for 28 cycles as follows: 10 seconds at 96°C, 5 seconds at 50°C and 30 seconds at 60°C. SNaPshot products were then incubated at 37°C for 1h with 0.8 U of thermo sensitive alkaline phosphatase (ThermoScientific^™^ FastAP^™^). After heat inactivation of the alkaline phosphatase for 20 minutes at 80°C, 1μL of the labeled products were mixed with 9.5 μL of HiDi^*™*^ formamide and 0.5 μL of GeneScan-120 LIZ size standard (Applied Biosystems). They were then separated using an ABIPRISM^®^ 3130xl Genetic Analyzer (Applied Biosystems) with POP-4 ^*™*^polymer and with respective run parameters: injection voltage of 1.2 Kv, injection time of 23 seconds run voltage of 15Kv and run time 1200 seconds in a capillary of 36 cm length. Analysis was performed using Gene Mapper ID software version 3.2.1 (Applied Biosystems). In order to confirm the genotyping system at least 10% of samples were sequenced on ABIPRISM^®^ 3130xl Genetic Analyzer (Applied Biosystems).

### Statistical Analysis

All statistical analysis was performed using Genetics package—software R version 2.11.1 [31]. Genotype frequencies were tested for Hardy Weinberg Equilibrium (HWE) using a Chi-square test. Frequencies of the genotypes, alleles and carriers were compared in cases and controls by logistic regression with adjustment for gender, income and age (> or < 25 years old). Income was included as a variable for correction since it was associated with TB outcome and could also be associated with ancestry (genetic variability). Categorical age criteria have been set according epidemiological features of TB [32]. Odds ratio (OR) was used as the point estimates of risk and were calculated along with their 95% confidence intervals (CIs) and the significant p values was adjusted by false discovery rate (FDR) test.

## Results

### Characteristics of PTB patients and healthy controls

Three-hundred sixty six individuals of the metropolitan area of Porto Alegre, Brazil were included in the study (Table 1). This city it is ranked as the Brazilian capital with the highest incidence of TB [33]. The PTB group was comprised of 191 individuals, 56 females and 135 males, with a mean age of 42.46 (±14.57). Regarding ethnicity within the PTB group, 134 individuals reported themselves as white and 56 as non-whites. However, this covariate showed no association with outcome in logistic regression model, so it was excluded of the analysis. Fifty-six subjects were infected with MDR-TB and 42 had comorbidities such as asthma, diabetes, hepatitis C, HIV/AIDS or HCV+HIV. The healthy control group included 175 individuals, 81 females and 94 males, with a mean age of 36.64 (±14.59) and 133 individuals declared themselves as white and 42 as non-white.

**Table 1:** Characteristics of The Population Studied.

Characteristics	PTB (n = 191)	HC (n = 175)
Age*	42.46 (14.57)	36.64 (14.59)
**Mean (SD)**		
**Gender**		
**Female, n (frequency)**	56 (0.29)	81 (0.46)
**Male, n (frequency)**	135 (0.71)	94 (0.54)
Ethnicity**		
**White n (frequency)**	134 (0.71)	133 (0.76)
**Non-white n (frequency)**	56 (0.29)	42 (0.24)
Income***	484,6 (437,6)	788,5 (572,5)
**Mean (SD)**		
**BCG vaccine**	Yes	Yes
**Comorbidities**	42 (0.22)	0
**n (frequency)**		
**MTB**		
**MDR-TB n (frequency)**	56 (0.29)	NA
**Sensitive n (frequency)**	135 (0.71)	

PTB pulmonary tuberculosis, HC healthy controls, n number of individuals, SD standard deviation, NA not apply, MTB *Mycobacterium tuberculosis*, MDR-TB tuberculosis multidrug resistant, Income was represented as American dollar

* Information was missing for 21 PTB (0.11) and 1 HC (0.006)

** Information was missing for 1 PTB (0.005)

*** Information was missing for 61 PTB (0.32) and 18 HC (0.10)

To confirm whether we could run our analysis combining household contacts and blood bank donors as one group defined as healthy controls, we first compared all SNPs in both populations and no difference was observed (S2 Table). Likewise, we compared patients infected with either drug sensitive or resistant strains (S3 Table) and no differences were observed. Thus, all following analyses were run comparing two groups: a combined PTB group and a combined healthy control group.

Cases and controls were in accordance with HWE (data not shown) for the SNPs evaluated except for the *IL17A* -692 C>T that deviated from HWE in cases and controls and *IFNG* +874T>A in control group. In order to avoid bias in our analysis *IL17A* -692 C>T and *IFNG* +874T>A were excluded of this study.

### SNPs in *IL17A* -197G>A and *IL6* -174G>A are associated with protection to PTB development

As observed in Table 2, for—*IL17A* 197A>G polymorphism the presence of -197A allele was significantly higher in control group than PTB patients (p = 0.04). Furthermore, the logistic regression model showed a decreased risk for PTB development among individuals with AA genotype (adjusted OR = 0.12; p = 0.04) and carriers of A allele (AG+AA) (adjusted OR = 0.29; p = 0.004).

**Table 2:** Distribution of *IL17A* -197G>A and *IL6* -174G>C Alleles, Genotypes and Carrier Frequencies in PTB Patients and Healthy Controls.

Gene/ refSNP	Allele/	PTB	HC	OR	p value	OR	p value
	Genotype	(n)	(n)	(95% CI)		(95% CI)*	
***IL17A***		171	133				
**rs2275913**	Allele G	307 (0.90)	214 (0.80)	Reference		Reference	
	Allele A	35 (0.10)	52 (0.20)	0.47 (0.24–0.90)	0.08	**0.29 (0.12–0.68)**	**0.04**
	GG	141 (0.82)	89 (0.67)	Reference		Reference	
	GA	25 (0.15)	36 (0.27)	**0.44 (0.24–0.78)**	**0.04**	0.35 (0.16–0.73)	0.20
	AA	5 (0.03)	8 (0.06)	0.39 (0.12–1.24)	0.44	**0.12 (0.02–0.64)**	**0.04**
	Carrier A	30 (0.18)	44 (0.33)	**0.43 (0.25–0.78)**	**0.008**	**0.29 (0.14–0.58)**	**0.004**
***IL6***		182	164				
**rs1800795**	Allele G	309 (0.85)	243 (0.74)	Reference		Reference	
	Allele C	55 (0.15)	85 (0.26)	0.51 (0.30–0.87)	0.08	0.46 (0.23–0.94)	0.12
	GG	133 (0.73)	94 (0.57)	Reference		Reference	
	GC	43 (0.24)	55 (0.34)	**0.55 (0.34–0.89)**	**0.04**	0.54 (0.29–1.00)	0.20
	CC	6 (0.03)	15 (0.09)	0.28 (0.10–0.75)	0.08	0.23 (0.06–0.89)	0.06
	Carrier C	49 (0.27)	70 (0.43)	**0.49 (0.31–0.78)**	**0.008**	**0.46 (0.26–0.83)**	**0.04**

PTB pulmonary tuberculosis, HC healthy controls, n number of individuals, OR odds ratio, CI confidence interval, *Logistic regression model adjusted by gender, age (> or < 25 years old) and income. p values was adjusted by FDR test. Bold values express statistically significant results, p < 0,05.

Regarding *IL6* -174G>C polymorphism (Table 2), the CG genotype and carriers of C allele (CC+CG) exhibited a decreased risk of PTB development (OR = 0.55, p = 0.04 and OR = 0.49, p = 0.008 respectively). Heterozygotes for -174 (GC) became not statistically significant after statistical adjustment (OR = 0.54, p = 0.20),

Taken together our results suggest that polymorphisms of *IL17A* and *IL6* cytokine genes at positions -197 and -174, respectively, are associated with protection for PTB development in the Southern Brazilian population.

### SNPs in *IL2–*330 T>G, *IL4*–590 C>T, *IL10*–592 A>C, *IL10*–1082 G>A, *TNF* -238 G>A and -308 G>A are not associated with to PTB development

Among polymorphisms at positions *IL2–*330 T>G, *IL4*–590 C>T, *IL10*–592 A>C, *IL10*–1082 G>A, *TNF* -238 G>A and *TNF* -308 G>A were found no evidence of association for genotypes, alleles and allele carriers and PTB development (S4 Table). Thus, our results suggest that the SNPs of cytokine genes *IL2*, *IL4*, *IL10* and *TNF* above are not directly related to PTB outcome.

## Discussion

Genetic effect on TB susceptibility is more complicated than many other common human diseases because there are potential confounding environmental factors involved in TB outcome [34]. However, considerable evidence has accumulated to support the role of human genetic factors in TB susceptibility [7–9]. In the past few years, population case-control studies have reported association between genetic variations in immune-related genes and susceptibility to TB [18, 20]. SNPs in cytokine genes are able to influence the cytokine levels and regulate resistance and susceptibility to TB [35,36].

The IL-17 cytokine family includes several cytokines among which IL-17A is thought a main pro-inflammatory cytokine being important to the innate and adaptive immune responses [37]. Recent progress in studies of IL-17A cytokine has revealed its important role in protective mechanisms against infectious diseases [24, 25, 38] and in studies of BCG-induced immunity [39–40].

The present study shows association between the -197G>A polymorphism and PTB susceptibility in Southern Brazilian population. Our results revealed that -197A allele that was associated with TB, control higher levels of IL-17A release, regulating promoter activity through affinity to transcriptional factor NFAT [41, 42]. Therefore, increased production of IL-17A could be beneficial in to hamper the mycobacterial infection contributing in formation of mature granuloma and blocking disease progression [43, 44].

The *IL17A* polymorphism (rs2275913) was also evaluated in distinct populations reporting contradictory results. In Northern Spain population, the G allele (OR = 1.40, p = 0.02) and GG genotype (OR = 1.59, p = 0.015) were associated with increased risk for PTB development [45] whereas two other studies failed in replicate such association in Croatian and a Chinese populations [46, 47]. In contrast, a recent report showed A allele carrier (GA/AA) (OR = 1.52, p = 0.006) and AA genotype (OR = 2.2, p = 0.001) associated to increased risk for PTB development [48]. However, this latter study did not fulfill HWE criteria for cases. Therefore, these results should be evaluated with caution. Our findings are converging with the literature suggesting the A allele of *IL17A* (rs2275913) SNP is associated with TB protection or G allele is associated with TB susceptibility, which means rigorously the same.

Interestingly, the A allele of *IL17A* (rs2275913) has been associated with increased risk for inflammatory diseases [49,50] indicating that its overproduction could as well be deleterious leading to pathological conditions. However, in MTB infection is likely that a genetic variation slightly increasing IL-17A production may be advantageous in the formation of mature granulomas making difficult mycobacteria spread [44].

The importance of cytokine IL-6 have been shown studies murine models of mycobacterial infections as well in vaccination with a tuberculosis subunit vaccine demonstrating that IL-6 is needed for optimal T-cell development [51, 52]. Indeed, high levels of IL-6 cytokine are produced in response to MTB infection [53, 54] and its role seems especially critical when bacterial burden is high [55]. Reports about the involvement of polymorphic variants within *IL6* gene and PTB susceptibility remain inconsistent [56]. To provide further investigation into controversial points between *IL6* polymorphisms and TB susceptibility we analyzed the influence of *IL6*–174 G>C polymorphism on PTB outcome. Our results suggested decreased risk for PTB development among carrier of C allele. The results corroborated previous findings among Pakistani population and confirmed by meta-analysis study [56, 57]. It was previously demonstrated that -174G allele is associated with higher production levels of IL-6 [58]. In this context, it was recognized that during MTB infection higher levels of IL-6 affect the production of other critical cytokines of immune response, such as IFN-γ [59]. Therefore, the decreased frequency of high IL-6 production genotype observed among the controls could be favorable for optimal macrophage activation and effective immunity in mycobacterial infection.

In contrast, the present study showed that *IL2*–330 T>G is not associated with PTB susceptibility, although the role of IL-2 cytokine in modulating the immune response in TB has become evident. Similarly, it was found no evidence of association between PTB and *IL4* -590C>T, *TNF* -308G>A and *TNF* -238G>A polymorphisms. Our results corroborate with previous reports concerning TB susceptibility in non-Brazilian populations [18, 60, 61], although, recently the -308A was associated with PTB susceptibility on an ethnically distinct population in Mozambique [62]. Also, the IL4 -590CC genotype showed a protective effect for PTB in a Southern India population [63]. Concerning our results for *IL10–*1082 and -592, we found no influence of these polymorphisms on PTB outcome. Our result for *IL10* polymorphism at -1082 position corroborates with a meta-analysis study in a worldwide-pooled population [18].

Interestingly, more recent meta-analysis studies that performed subgroup analysis for the *IL10* -1082G>A SNP revealed conflicting results for analysis stratified by ethnicity for European and American population [20, 64]. Further, according to our findings, *IL10*–592 A>C analysis showed no risk association with PTB and a meta-analysis study confirmed such finding for Europeans, Africans, and Americans populations but reported a decreased risk for TB development among the Asian population [20]. In this context, reports have highlighted the importance of haplotype analysis since both polymorphisms -592 A>C and -819 C>T in *IL10* promoter region are in perfect linkage disequilibrium [64, 65].

The use of popular case-control designs to test candidate genes or genome-wide association studies have proved suitable to pinpoint functional genetic variants in complex diseases [66]. Sometimes it is necessary to test several independent population to replicate the data, but results could be useful to envisage better diagnostics and prognostics methods [66, 67]. TB used to be a chronic disease and it is likely that a subtle balance of immune response could have a great impact over its control and progression. In this regard, our study showed for the first time in that population the protective role of *IL17A* -197G>A and *IL6* -174G>C on PTB outcome. Indeed, our findings support *IL17A* (rs2275913) and *IL6* (rs1800795) polymorphisms as promising markers for PTB risk and indicate the role of IL-6 and IL-17A cytokines in immunophysiology of TB. This novel information of host response to MTB infection can help design more effective vaccines, identify risk populations and new treatment strategies. In the future, additional larger studies are needed to validate our findings. Moreover, a dense SNP mapping on *IL6* and *IL17A* loci could be interestingly for better elucidate such association.

## Supporting Information

S1 Table Primer Sequences for Conventional PCR and for SNaPshot Multiplex System. (DOCX)

S2 Table Allelic and Genotypic Frequencies for Cytokine SNPs in Blood Donors and Household Contacts. (DOCX)

S3 Table Allelic and Genotypic Frequencies for Cytokine SNPs in Pulmonary Tuberculosis Patients Sensitive and Multidrug-Resistant Infected. (DOCX)

S4 Table Allelic, Genotypic and Carrier Frequencies of Cytokine SNPs in Pulmonary Tuberculosis Cases and Healthy Controls. (DOCX)
