# Supplementary material for: Single Nucleotide Polymorphisms in IL17A and IL6 Are Associated with Decreased Risk for Pulmonary Tuberculosis in Southern Brazilian Population
Source: PLoS One. 2016 Feb 3;11(2):e0147814. doi: 10.1371/journal.pone.0147814 (PMC4740512; doi:10.1371/journal.pone.0147814)
Supplement: S1 Table — (DOCX) [file pone.0147814.s001.docx]

**S1 Table. Primer Sequences for Conventional PCR and for SNaPshot Multiplex System.**

| **_refSNP_** | **_Primer Sequence_** |
| --- | --- |
| **_rs1800896_** | _F 5' - ttccccaggtagagcaacac- 3'/ R 5' -atggaggctggataggaggt - 3'_  _aaatccaagacaacactactaaggcttctttgggaA/G_ |
| **_rs8193036_** | _F 5' - ccttctctctttcccccatc- 3' / R 5' - tgcatgctaccaagcaactt- 3'_  _catcactctctactcccccctgccccccttttctccatctC/T_ |
| **_rs2243250_** | _F 5' - acccaaactaggcctcacct- 3' / R 5' - acaggtggcatcttggaaac- 3'_  _gatacgacctgtccttctcaaaacacctaaacttgggagaacattgtC/T_ |
| **_rs1800872_** | _F 5' - ggggtcatggtgagcactac- 3' / R 5' - caagcagcccttccatttta- 3'_  _atcctaatgaaatcggggtaaaggagcctggaacacatcctgtgaccccgcctgtA/C_ |
| **_rs1800629_** | _F 5' - gcccctcccagttctagttc- 3' / R 5' - aaagttggggacacacaagc- 3'_  _aggaaacagaccacagacctggtccccaaaagaaatggaggcaataggttttgaggggcatgA/G_ |
| **_rs2069762_** | _F 5' - ccattctgaaacaggaaacca- 3' / R 5' - aaacccccaaagactgactg- 3'_  _ttattcttttcatctgtttactcttgctcttgtccaccacaatatgctattcacatgttcagtgtagttttaG/T_ |
| **_rs2430561_** | _F 5' -gcaaagccaccccactataa - 3' / R 5' -cttcgttgctcactggggatt - 3'_  _tcctgtagggtattattatacgagctttaaaagatagttccaaacatgtgcgagtgtgtgtgtgtgtgtgtgtgtgtgtgT/A_ |
| **_rs1800795_** | _F 5' - tcgtgcatgacttcagcttt- 3' / R 5' - gcctcagacatctccagtcc- 3'_  _aaagaaagtaaaggaagagtggttctgcttcttagcgctagcctcaatgacgacctaagctgcacttttccccctagttgtgtcttgcC/G_ |
| **_rs2275913_** | _F 5' - gccaaggaatctgtgaggaa- 3' / R 5' -ttcaggggtgacaccatttt - 3'_  _gcatagcagctctgctcagcttctaacaagtaagaatgaaaagaggacatggtctttaggaacatgaatttctgcccttcccattttccttcagaagA/G_ |
| **_rs361525_** | _F 5' - gcccctcccagttctagttc- 3' / R 5' - aaagttggggacacacaagc- 3'_  _aaaagaaatggaggcaataggttttgaggggcatggggacggggttcagcctccagggtcctacacacaaatcagtcagtggcccagaagacccccctcggaatcA/G_ |
