# Supplementary material for: Single Nucleotide Polymorphisms in IL17A and IL6 Are Associated with Decreased Risk for Pulmonary Tuberculosis in Southern Brazilian Population
Source: PLoS One. 2016 Feb 3;11(2):e0147814. doi: 10.1371/journal.pone.0147814 (PMC4740512; doi:10.1371/journal.pone.0147814)
Supplement: S2 Table — (DOCX) [file pone.0147814.s002.docx]

**S2 Table. Allelic and Genotypic Frequencies for Cytokine SNPs in Blood Donors and Household Contacts.**

| **_Gene/ refSNP_** | **_Allele/_**  **_Genotype_** | **_Blood Donor (n)_** | **_Household Contact (n)_** | **_OR (p valor)_** |
| --- | --- | --- | --- | --- |
| **_IL-2_** |  | _119_ | _46_ |  |
| **_rs2069762_** | _Allele T_ | _156 (0.66)_ | _69 (0.75)_ | _Reference_ |
|  | _Allele G_ | _82 (0.34)_ | _23 (0.25)_ | _0.63 (0.24)_ |
|  | _TT_ | _57 (0.48)_ | _23 (0.5)_ | _Reference_ |
|  | _TG_ | _42 (0.35)_ | _23 (0.5)_ | _1.35 (0.39)_ |
|  | _GG_ | _20 (0.17)_ | _0_ | _0_ |
| **_IL-4_** |  | _123_ | _49_ |  |
| **_rs2243250_** | _Allele C_ | _169 (0.69)_ | _63 (0.64)_ | _Reference_ |
|  | _Allele T_ | _77 (0.31)_ | _35 (0.36)_ | _1.22 (0.57)_ |
|  | _CC_ | _58 (0.47)_ | _21 (0.43)_ | _Reference_ |
|  | _TC_ | _53 (0.43)_ | _21 (0.43)_ | _1.09 (0.80)_ |
|  | _TT_ | _12 (0.10)_ | _7 (0.14)_ | _1.61 (0.38)_ |
| **_IL-6_** |  | _119_ | _45_ |  |
| **_rs1800795_** | _Allele G_ | _170 (0.71)_ | _73 (0.81)_ | _Reference_ |
|  | _Allele C_ | _68 (0.29)_ | _17 (0.19)_ | _0.58 (0.21)_ |
|  | _GG_ | _64 (0.54)_ | _30 (0.67)_ | _Reference_ |
|  | _GC_ | _42 (0.35)_ | _13 (0.29)_ | _0.66 (0.28)_ |
|  | _CC_ | _13 (0.11)_ | _2 (0.04)_ | _0.33 (0.16)_ |
| **_IL-10_** |  | _123_ | _49_ |  |
| **_rs1800872_** | _Allele C_ | _172 (0.70)_ | _67 (0.68)_ | _Reference_ |
|  | _Allele A_ | _74 (0.30)_ | _31 (0.32)_ | _1.07 (0.84)_ |
|  | _CC_ | _55 (0.45)_ | _24 (0.49)_ | _Reference_ |
|  | _AC_ | _62 (0.50)_ | _19 (0.39)_ | _0.70 (0.32)_ |
|  | _AA_ | _6 (0.05)_ | _6 (0.12)_ | _2.29 (0.19)_ |
| **_IL-10_** |  | _123_ | _49_ |  |
| **_rs1800896_** | _Allele A_ | _161 (0.65)_ | _63 (0.64)_ | _Reference_ |
|  | _Allele G_ | _85 (0.35)_ | _35 (0.36)_ | _1.05 (0.88)_ |
|  | _AA_ | _48 (0.39)_ | _21 (0.43)_ |  |
|  | _AG_ | _65 (0.53)_ | _21 (0.43)_ | _0.74 (0.40)_ |
|  | _GG_ | _10 (0.08)_ | _7 (0.14)_ | _1.6 (0.40)_ |
| **_IL-17A_** |  | _101_ | _32_ |  |
| **_rs2275913_** | _Allele G_ | _160 (0.79)_ | _54 (0.84)_ | _Reference_ |
|  | _Allele A_ | _42 (0.21)_ | _10 (0.16)_ | _0.70 (0.52)_ |
|  | _GG_ | _66 (0.65)_ | _23 (0.72)_ | _Reference_ |
|  | _AG_ | _28 (0.28)_ | _8 (0.25)_ | _0.82 (0.67)_ |
|  | _AA_ | _7 (0.07)_ | _1 (0.03)_ | _0.40 (0.41)_ |
| **_TNF_** |  | _122_ | _49_ |  |
| **_rs1800629_** | _Allele G_ | _221 (0.9)_ | _85 (0.87)_ | _Reference_ |
|  | _Allele A_ | _25 (0.1)_ | _13 (0.13)_ | _1.35 (0.56)_ |
|  | _GG_ | _100 (0.81)_ | _38 (0.78)_ | _Reference_ |
|  | _AG_ | _21 (0.17)_ | _9 (0.18)_ | _1.12 (0.78)_ |
|  | _AA_ | _2 (0.02)_ | _2 (0.04)_ | _2.63 (0.34)_ |
| **_TNF_** |  | _123_ | _49_ |  |
| **_rs361525_** | _Allele G_ | _238 (0.97)_ | _92 (0.94)_ | _Reference_ |
|  | _Allele A_ | _8 (0.03)_ | _6 (0.06)_ | _1.94 (0.40)_ |
|  | _GG_ | _115 (0.93)_ | _44 (0.90)_ | _Reference_ |
|  | _AG_ | _8 (0.07)_ | _4 (0.08)_ | _1.31 (0.67)_ |
|  | _AA_ | _0_ | _1 (0.02)_ | _0_ |
