# Supplementary material for: Single Nucleotide Polymorphisms in IL17A and IL6 Are Associated with Decreased Risk for Pulmonary Tuberculosis in Southern Brazilian Population
Source: PLoS One. 2016 Feb 3;11(2):e0147814. doi: 10.1371/journal.pone.0147814 (PMC4740512; doi:10.1371/journal.pone.0147814)
Supplement: S3 Table — (DOCX) [file pone.0147814.s003.docx]

**S3 Table. Allelic and Genotypic Frequencies for Cytokine SNPs in Pulmonary Tuberculosis Patients Sensitive and Multidrug-Resistant Infected.**

| **_Gene/ refSNP_** |  | **_Sensitive_** | **_MDR_** | **_OR (p valor)_** | **_p value*_** |
| --- | --- | --- | --- | --- | --- |
| **_IL-2_** |  | _100_ | _55_ |  |  |
| **_rs2069762_** | _Allele T_ | _134 (0.67)_ | _72 (0.68)_ | _Reference_ |  |
|  | _Allele G_ | _66 (0.33)_ | _34 (0.32)_ | _0.96 (0.91)_ |  |
|  | _TT_ | _46 (0.46)_ | _26 (0,47)_ | _Reference_ |  |
|  | _TG_ | _42 (0.42)_ | _24 (0.45)_ | _1.09 (0,80)_ |  |
|  | _GG_ | _12 (0.12)_ | _5 (0.09)_ | _0.80 (0.70)_ |  |
| **_IL-4_** |  | _100_ | _54_ |  |  |
| **_rs2243250_** | _Allele C_ | _125 (0.62)_ | _67 (0.62)_ | _Reference_ |  |
|  | _Allele T_ | _75 (0.38)_ | _41 (0.38)_ | _1.02 (0.95)_ |  |
|  | _CC_ | _37 (0.37)_ | _22 (0.41)_ | _Reference_ |  |
|  | _TC_ | _51 (0.51)_ | _23 (0.43)_ | _0.76 (0.45)_ |  |
|  | _TT_ | _12 (0.12)_ | _9 (0.16)_ | _1.26 (0.65)_ |  |
| **_IL-6_** |  | _99_ | _54_ |  |  |
| **_rs1800795_** | _Allele G_ | _167 (0.84)_ | _95 (0.88)_ | _Reference_ |  |
|  | _Allele C_ | _31 (0.16)_ | _13 (0.12)_ | _0.74 (0.54)_ |  |
|  | _GG_ | _71 (0.72)_ | _43 (0.80)_ | _Reference_ |  |
|  | _GC_ | _25 (0.24)_ | _9 (0.17)_ | _0.59 (0.23)_ |  |
|  | _CC_ | _3 (0.03)_ | _2 (0.04)_ | _1.10 (0.91)_ |  |
| **_IL-10_** |  | _100_ | _54_ |  |  |
| **_rs1800872_** | _Allele C_ | _136 (0.68)_ | _66 (0.61)_ | _Reference_ |  |
|  | _Allele A_ | _64 (0.32)_ | _42 (0.39)_ | _1.35 (0.38)_ |  |
|  | _CC_ | _44 (0.44)_ | _20 (0.37)_ | _Reference_ |  |
|  | _AC_ | _48 (0.48)_ | _26 (0.48)_ | _1.19 (0.63)_ |  |
|  | _AA_ | _8 (0.08)_ | _8 (0.15)_ | _2.2 (0.16)_ |  |
| **_IL-10_** |  | _100_ | _54_ |  |  |
| **_rs1800896_** | _Allele A_ | _133 (0.66)_ | _70 (0.65)_ | _Reference_ |  |
|  | _Allele G_ | _67 (0.34)_ | _38 (0.35)_ | _1.07 (0.83)_ |  |
|  | _AA_ | _43 (0.43)_ | _23 (0.43)_ | _Reference_ |  |
|  | _AG_ | _47 (0.47)_ | _24 (0.44)_ | _0.95 (0.90)_ |  |
|  | _GG_ | _10 (0.10)_ | _7 (0.13)_ | _1.30 (0.63)_ |  |
| **_IL-17A_** |  | _97_ | _51_ |  |  |
| **_rs2275913_** | _Allele G_ | _179 (0.92)_ | _95 (0.93)_ | _Reference_ |  |
|  | _Allele A_ | _15 (0.08)_ | _7 (0.07)_ | _0.88 (0.85)_ |  |
|  | _GG_ | _84 (0.87)_ | _44 (0.86)_ | _Reference_ |  |
|  | _AG_ | _11 (0.11)_ | _7 (0.14)_ | _1.21 (0.70)_ |  |
|  | _AA_ | _2 (0.02)_ | _0_ | _-_ |  |
| **_TNF_** |  |  |  |  |  |
| **_rs361525_** | _Allele G_ | _192 (0.97)_ | _94 (0.9)_ | _Reference_ |  |
|  | _Allele A_ | _6 (0.03)_ | _10 (0.1)_ | _3.04 (0.10)_ |  |
|  | _GG_ | _93 (0.94)_ | _42 (0.81)_ | _Reference_ |  |
|  | _AG_ | _6(0.06)_ | _10 (0.19)_ | _3.69 (0,02)_ | _0.16_ |
| **_TNF_** |  | _100_ | _53_ |  |  |
| **_rs1800629_** | _Allele G_ | _173(0.86)_ | _94(0.89)_ | _Reference_ |  |
|  | _Allele A_ | _27 (0.14)_ | _12 (0.11)_ | _0.82 (0.70)_ |  |
|  | _GG_ | _73 (0.73)_ | _41 (0.77)_ | _Reference_ |  |
|  | _AG_ | _27(0.27)_ | _12 (0.23)_ | _0.79 (0.55)_ |  |
