# Supplementary material for: Single Nucleotide Polymorphisms in IL17A and IL6 Are Associated with Decreased Risk for Pulmonary Tuberculosis in Southern Brazilian Population
Source: PLoS One. 2016 Feb 3;11(2):e0147814. doi: 10.1371/journal.pone.0147814 (PMC4740512; doi:10.1371/journal.pone.0147814)
Supplement: S4 Table — (DOCX) [file pone.0147814.s004.docx]

**S4 Table. Allelic, Genotypic and Carrier Frequencies of Cytokine SNPs in Pulmonary Tuberculosis Cases and Healthy Controls.** *Logistic regression model adjusted by gender, age (> or < 25 years old) and income (numerical variable), *p values was adjusted by FDR test

| **_Gene/ refSNP_** | **_Allele/_**  **_Genotype_** | **_PTB (n)_** | **_HC (n)_** | **_OR (95% CI)_** | **_p value_** | **_OR (95% CI)*_** | **_* p value_** |
| --- | --- | --- | --- | --- | --- | --- | --- |
| **_IL-2_** |  | _181_ | _165_ |  |  |  |  |
| **_rs2069762_** | _Allele T_ | _248 (0.69)_ | _225 (0.68)_ | _Reference_ |  | _Reference_ |  |
|  | _Allele G_ | _114 (0.31)_ | _105 (0.32)_ | _0.98 (0.62-1.55)_ | _0.95_ | _1.09 (0.62-1.91)_ | _0.75_ |
|  | _TT_ | _86 (0.48)_ | _80 (0.48)_ | _Reference_ |  | _Reference_ |  |
|  | _TG_ | _76 (0.42)_ | _65 (0.39)_ | _1.08 (0.69-1.70)_ | _0.71_ | _1.42 (0.81-2.48)_ | _0.22_ |
|  | _GG_ | _19 (0.10)_ | _20 (0.12)_ | _0.88 (0.44-1.77)_ | _0.73_ | _0.90 (0.37-2.20)_ | _0.82_ |
|  | _Carrier G_ | _162 (0.85)_ | _145 (0.88)_ | _1.04 (0.68-1.59)_ | _0.86_ | _1.30 (0.77-2.19)_ | _0.33_ |
| **_IL-4_** |  | _183_ | _172_ |  |  |  |  |
| **_rs2243250_** | _Allele C_ | _239 (0.65)_ | _232 (0.67)_ | _Reference_ |  | _Reference_ |  |
|  | _Allele T_ | _127 (0.35)_ | _112 (0.33)_ | _1.1 (0.7-1.71)_ | _0.67_ | _1.29 (0.74-2.22)_ | _0.36_ |
|  | _CC_ | _79 (0.43)_ | _79 (0.46)_ | _Reference_ |  | _Reference_ |  |
|  | _TC_ | _81 (0.44)_ | _74 (0.43)_ | _1.09 (0.70-1.70)_ | _0.69_ | _1.49 (0.86-2.59)_ | _0.16_ |
|  | _TT_ | _23 (0.13)_ | _19 (0.11)_ | _1.21 (0.61-2.39)_ | _0.58_ | _1.42 (0.61-3.29)_ | _0.42_ |
|  | _Carrier T_ | _160 (0.87)_ | _153 (0.89)_ | _1.11 (0.73-1.70)_ | _0.60_ | _1.47 (0.87-2.48)_ | _0.14_ |
| **_IL-10_** |  | _183_ | _172_ |  |  |  |  |
| **_rs1800872_** | _Allele C_ | _240 (0.66)_ | _239 (0.69)_ | _Reference_ |  | _Reference_ |  |
|  | _Allele A_ | _126 (0.34)_ | _105 (0.31)_ | _1.19 (0.76-1.86)_ | _0.43_ | _1.25 (0.72-2.15)_ | _0.43_ |
|  | _CC_ | _75 (0.41)_ | _79 (0.46)_ | _Reference_ |  | _Reference_ | _Reference_ |
|  | _AC_ | _90 (0.49)_ | _81 (0.47)_ | _1.17 (0.76-1.80)_ | _0.46_ | _1.29 (0.75-2.20)_ | _0.36_ |
|  | _AA_ | _18 (0.10)_ | _12 (0.07)_ | _1.58 (0.71-3.50)_ | _0.26_ | _1.63 (0.62-4.32)_ | _0.32_ |
|  | _Carrier A_ | _108 (0.59)_ | _93 (0.48)_ | _1.22 (0.80-1.86)_ | _0.35_ | _1.33 (0.79-2.24)_ | _0.28_ |
| **_IL-10_** |  | _183_ | _172_ |  |  |  |  |
| **_rs1800896_** | _Allele A_ | _242 (0.66)_ | _224 (0.65)_ | _Reference_ |  | _Reference_ |  |
|  | _Allele G_ | _124 (0.34)_ | _120 (0.35)_ | _0.96 (0.61-1.48)_ | _0.84_ | _0.87 (0.50-1.49)_ | _0.61_ |
|  | _AA_ | _79 (0.43)_ | _69 (0.4)_ | _Reference_ |  | _Reference_ |  |
|  | _AG_ | _84 (0.46)_ | _86 (0.5)_ | _0.85 (0.55-1.32)_ | _0.48_ | _0.73 (0.42-1.26)_ | _0.26_ |
|  | _GG_ | _20 (0.11)_ | _17 (0.1)_ | _1.02 (0.50-2.12)_ | _0.94_ | _0.89 (0.36-2.24)_ | _0.81_ |
|  | _Carrier G_ | _104 (0.57)_ | _103 (0.60)_ | _0.88 (0.57-1.34)_ | _0.56_ | _0.75 (0.45-1.27)_ | _0.29_ |
| **_TNF_** |  | _180_ | _172_ |  |  |  |  |
| **_rs361525_** | _Allele G_ | _342 (0.95)_ | _330 (0.96)_ | _Reference_ |  | _Reference_ |  |
|  | _Allele A_ | _18 (0.05)_ | _14 (0.04)_ | _1.24 (0.45-3.41)_ | _0.67_ | _0.91 (0.27-3.08)_ | _0.87_ |
|  | _GG_ | _162 (0.9)_ | _159 (0.92)_ | _Reference_ |  | _Reference_ |  |
|  | _AG_ | _18 (0.1)_ | _12 (0.07)_ | _1.47 (0.68-3.15)_ | _0.32_ | _1.09 (0.43-2.76)_ | _0.86_ |
|  | _AA_ | _0_ | _1 (0.003)_ | _NA_ | _NA_ | _NA_ | _NA_ |
|  | _Carrier A_ | _18 (0.1)_ | _13 (0.08)_ | _1.36 (0.64-2.86)_ | _0.42_ | _0.99 (0.72-2.49)_ | _0.98_ |
| **_TNF_** |  | _182_ | _172_ |  |  |  |  |
| **_rs1800629_** | _Allele G_ | _322 (0.88)_ | _306 (0.89)_ | _Reference_ |  | _Reference_ |  |
|  | _Allele A_ | _42 (0.12)_ | _38 (0.11)_ | _1.05 (0.54-2.02)_ | _0.88_ | _1.12 (0.50-2.48)_ | _0.78_ |
|  | _GG_ | _140 (0.77)_ | _138 (0.8)_ | _Reference_ |  | _Reference_ |  |
|  | _AG_ | _42 (0.23)_ | _30 (0.17)_ | _1.38 (0.81-2.33)_ | _0.23_ | _1.54 (0.82-2.93)_ | _0.18_ |
|  | _AA_ | _0_ | _4 (0.02)_ | _NA_ | _NA_ | _NA_ | _NA_ |
|  | _Carrier A_ | _42 (0.23)_ | _34 (0.20)_ | _1.21 (0.73-2.02)_ | _0.45_ | _1.34 (0.72-2.49)_ | _0.35_ |
